# Detection of Renal Injury Following Primary Coronary Intervention among ST-Segment Elevation Myocardial Infarction Patients: Doubling the Incidence Using Neutrophil Gelatinase-Associated Lipocalin as a Renal Biomarker

**DOI:** 10.3390/jcm10102120

**Published:** 2021-05-14

**Authors:** Lior Lupu, Keren-Lee Rozenfeld, David Zahler, Samuel Morgan, Ilan Merdler, Moshe Shtark, Ilana Goldiner, Shmuel Banai, Yacov Shacham

**Affiliations:** Department of Cardiology, Tel-Aviv Sourasky Medical Center affiliated to the Sackler Faculty of Medicine, Tel-Aviv University, Tel-Aviv 64239, Israel; Liorlupu@gmail.com (L.L.); kerenlees@gmail.com (K.-L.R.); david.zahler@gmail.com (D.Z.); samuelmorgan@mail.tau.ac.il (S.M.); Ilanmerdler@gmail.com (I.M.); Moshes@tlvmc.gov.il (M.S.); Ilanag@tlvmc.gov.il (I.G.); shmuelb@tlvmc.gov.il (S.B.)

**Keywords:** neutrophil gelatinase-associated lipocalin, ST-segment elevation myocardial infarction, renal injury

## Abstract

Background: A subgroup of patients with acute kidney injury (AKI) do not fulfil the functional criteria for AKI diagnosis but show elevated levels of new biomarkers reflecting tubular injury, suggesting that these patients suffer “subclinical AKI”. We investigated the incidence and possible implications of “subclinical AKI”, compared to no and clinical AKI among ST elevation myocardial infarction patients (STEMI) treated with primary coronary intervention (PCI). Methods: We included 223 patients with STEMI treated with PCI. Neutrophil gelatinase-associated lipocalin (NGAL) was used as a marker of renal tubular damage in the absence of functional AKI, with NGAL levels ≥100 ng/mL suggesting subclinical AKI. Patients were assessed for the occurrence of in-hospital adverse outcomes. Results: Of the study patients, 45 (25%) had subclinical AKI. These patients were more likely to have left ventricular ejection fraction ≤45% (33% vs. 23%. *p* = 0.01), in-hospital adverse outcomes (73% vs. 48%; *p* = 0.005), and a combination of the two. The multivariate regression model demonstrated that subclinical AKI was independently associated with in-hospital adverse outcomes (OR 3.71, 95% CI 1.30–10.62, *p* = 0.02). Conclusions: Subclinical AKI is common among STEMI patients and is independently associated with adverse outcomes, even in the absence of functional AKI.

## 1. Introduction

According to consensus criteria, identification of acute kidney injury (AKI) is based on serum creatinine (sCr) changes. However, changes in sCr merely reflect functional changes and do not incorporate parameters that directly indicate tubular damage. Furthermore, an acute change in glomerular function after a renal insult does not result in an abrupt increase in sCr due to the time needed for creatinine to accumulate in the body [1,2], and it usually takes 24–36 h after a renal insult to reach a new steady state [3]. Over the past decade, several novel biomarkers have been introduced, allowing earlier detection of tubular and glomerular injury when compared to sCr [4,5]. Based on these biomarkers, members of the Acute Dialysis Quality Initiative (ADQI) group proposed a reformulation of the diagnostic approach to AKI where not only markers of kidney function (e.g., changes in sCr and urine output), but also markers of kidney damage are incorporated. A subgroup of AKI patients was described who did not fulfil the classically used functional criteria for AKI diagnosis but showed elevated levels of new biomarkers presumably reflecting tubular injury, suggesting that these patients suffered from “subclinical AKI” [6]. Neutrophil gelatinase-associated lipocalin (NGAL) is a glycoprotein stored in granules of mature neutrophils in complex with gelatinase. It is reabsorbed by proximal tubules and released by damaged distal tubules in cases of acute tubular damage and may be detected within a few hours of the tubular insult and even in the absence of functional AKI [7,8,9,10,11,12]. A large proportion of patients admitted to hospital have various degrees of heart and kidney dysfunction. Bidirectional interactions exist between the heart and kidneys, defined as various types of cardiorenal syndrome (CRS), where a primary disorder of one of these organs often results in secondary dysfunction or injury to the other [13]. We investigated the incidence and possible implications of subclinical AKI among ST elevation myocardial infarction patients (STEMI) undergoing primary percutaneous coronary intervention (PCI).

## 2. Materials and Methods

### 2.1. Patients

A prospective, observational, open-label trial was performed in the Tel Aviv Sourasky medical center. Included were 223 STEMI patients admitted to the cardiac intensive care unit (CICU) following primary PCI for the diagnosis of STEMI between November 2017 to May 2020. Diagnosis of STEMI was established by a typical history of chest pain, diagnostic electrocardiographic changes, and serial elevation of serum cardiac biomarkers [14]. Primary PCI was performed in patients with symptoms ≤12 h in duration as well as in patients with symptoms lasting 12–24 h in duration if the symptoms continued to persist at the time of admission. The contrast medium used in procedures was iodixanol (Visipaque, GE healthcare, Ireland). Following coronary interventional procedures, physiologic (0.9%) saline was given intravenously at a rate of 1 mL/kg/h for 12 h after contrast exposure. In patients with overt heart failure, the hydration rate was reduced at the discretion of the attending physician. All patients underwent a screening echocardiographic examination within three days of admission to assess left ventricular (LV) ejection fraction. Patient records were evaluated for the in-hospital course and occurrence of adverse outcomes. These included the development of heart failure episodes (defined as the occurrence of both clinical and radiological signs of congestion) treated conservatively, respiratory failure with the need for mechanical ventilation, new onset atrial fibrillation episodes, as well as in-hospital mortality. These complications were united to form a composite outcome of major adverse in-hospital cardiac events. The combined endpoint was the presence of LV ejection fraction of ≤45% and/or major adverse in-hospital cardiac events. Informed consent was obtained from all individual participants included in the study. The study protocol was approved by the local institutional ethics committee (Institutional Board Review no.: TLV-16-0224).

### 2.2. Laboratory

Serum NGAL levels of venous blood were collected from all patients 24 h following admission to the CICU. Samples were centrifuged within 10 min in a refrigerated centrifuge, and the plasma and serum were stored at −80 °C. NGAL was analyzed using NGAL rapid Elisa kits (Bioporto Diagnostics, Copenhagen, Denmark). AKI was defined using the KDIGO criteria as an increase in serum creatinine of 0.3 mg/dL or more within 48 h, or an increase in serum creatinine above 1.5 times baseline within seven days of admission.

We applied the term subclinical AKI to indicate the absence or presence of tubular injury, in the absence of functional AKI, manifested as sCr elevation [1]. The presence of renal damage was defined according to the cardiac surgery-associated NGAL score (CSA-NGAL score) with NGAL levels >100 ng/mL suggesting subclinical AKI [15].

The sCr was determined upon hospital admission, prior to PCI, and at least once a day throughout hospitalization and was available for all analyzed patients. The estimated glomerular filtration rate (eGFR) was estimated using the Chronic Kidney Disease Epidemiology Collaboration (CKD-EPI) Equation [16]. Chronic kidney disease (CKD) was categorized as admission eGFR of <60 mL/min/1.73 m^2^ [17]. Blood samples for C-reactive protein (CRP) were drawn following primary PCI and within 24 h of CICU admission. Quantitative CRP analysis was performed by the Bayer wide-range assay. Patients were stratified into two groups based on the presence of subclinical AKI.

### 2.3. Statistics

All data are summarized and displayed as mean (±standard deviation) or median (interquartile range 25–75%) for continuous variables and as number (percentage) of patients in each group for categorical variables. Analysis of variance (ANOVA) test was performed for the comparison between patients having no AKI, subclinical AKI and clinical AKI, and post hoc analysis was performed between the groups. For post hoc analysis, two continuous variables were compared using the independent sample *t*-test for normally distributed data and the Mann–Whitney U test for non-normally distributed variables. Categorial variables were compared using the Chi square or Fisher exact test. The utilization of any and subclinical AKI to assess the risk for adverse in-hospital outcomes was evaluated using a multivariate logistic regression adjusted for all parameters found significant in the univariate analysis. An adjusted odds ratio (OR) with 95% confidence interval (CI) was reported for all variables. A two-tailed *p* value of <0.05 was considered significant for all analyses. All statistical analysis was performed using R statistical software (version 4.0.5; R Foundation for Statistical Computing, Vienna, Austria).

## 3. Results

The study population included 223 STEMI patients. The mean age was 62 ± 13 years, and 78% were men. Of these patients, 87 (39%) developed evidence of renal injury, of which 42 (19%) presented with AKI according to KDIGO and 45 (20%) with a subclinical tubular injury detected by NGAL.

Patients’ baseline characteristics according to AKI group are demonstrated in Table 1. Patients with AKI were older, with a lower baseline eGFR and longer symptom duration. In a post hoc analysis, the most prominent difference between patients with clinical AKI and subclinical AKI was the presence of any CKD (59% vs. 15%, *p* < 0.001) and stage 3a, 4, and 5 CKD (21% vs. 9%, *p* = 0.001). Table 2 presents the key in-hospital adverse outcomes of patients with vs. without subclinical AKI. Patients with subclinical AKI demonstrated longer length of stay, lower LV ejection fraction (44% vs. 47%, *p* = 0.06), and were more likely to have LV ejection fraction of ≤45% (33% vs. 23%, *p* = 0.01). In-hospital adverse outcomes were significantly higher among patients with subclinical AKI (73% vs. 48%, *p* = 0.005), with an odds ratio of 2.92 (95% CI 1.42–6.32, *p* = 0.005). The multivariate regression model (Table 3) demonstrated that, in addition to any AKI, subclinical AKI was independently associated with in-hospital adverse outcomes (OR 3.71; 95% CI 1.30–10.62, *p* = 0.02). Other factors associated with in-hospital adverse outcomes included peak CRP and left ventricular ejection fraction.

## 4. Discussion

Our study demonstrated that among STEMI patients treated with primary PCI, the presence of subclinical AKI (tubular damage biomarker positivity without loss of function) was common and associated with adverse in-hospital outcomes.

Clinical AKI is diagnosed when renal damage and dysfunction reach a threshold sufficient to make sCr rise above 0.3 mg/dL within 48 h or when oliguria is present for over 6 h [1]. sCr values are influenced by many non-renal determinants, and both the generation rate and the distribution volume of creatinine are not stable in critically ill patients. An acute change in GFR after a renal insult does not result in an abrupt increase in sCr due to the time needed for creatinine to accumulate in the body [2], and it usually takes 24–36 h after a renal insult to reach a new steady state [3]. The delayed increase in sCr after a decline in GFR is seen especially in patients who simultaneously become fluid overloaded. Similarly, sCr does not immediately decrease when GFR improves [18]. Lack of renal functional deterioration may also reflect the absence of subtle chronic kidney disease, with an intact renal functional reserve that compensates for a transient subtle injury [19]. Recent data suggest that early diagnosis of AKI can be made by using a single structural or functional biomarker (or a combination thereof) capable of detecting kidney injury almost in real time [6]. Many studies with several thousands of patients have shown evidence that there is an additional value to new biomarkers not only because they allow a diagnosis to be made earlier [7] but also because they allow a kidney injury to be diagnosed even in the absence of a subsequent manifest dysfunction [2,8]. Moreover, the fact that AKI is not clinically manifest does not necessarily mean that the kidney is intact and that the function is perfect. Indeed, patients with AKI were older, which may influence the eGFR values, which decrease with age, making them more vulnerable to renal damage.

NGAL is rapidly induced and released from the injured distal nephron in response to renal injury. Its urine and plasma concentrations increase proportionally to severity and duration of renal injury [20], and rapidly decrease with attenuation of renal injury [21]. In a pooled data analysis from 10 studies on patients admitted to intensive care units, Haase et al. demonstrated that elevated NGAL levels were associated with adverse outcomes even in the absence of diagnostic increases in sCr [22]. In another study, patients considered to have subclinical AKI (plasma NGAL positive/sCr negative) on admission at the emergency unit had significantly higher rates of clinical events for the combined endpoint of renal replacement therapy or in-hospital mortality than the patients who were sCr negative/plasma NGAL negative [23]. It is nevertheless possible that the adverse outcomes reflecting more severe cardiac injury and hemodynamic compromise may predispose hypoxic tubular injury. Thus, subtle kidney injury merely reflects a more severe multiorgan dysfunction affecting the kidney and the overall outcomes [24]. In the present cohort, patients with clinical AKI were more likely to have baseline CKD. Patients with CKD have limited functional reserve, hence less capacity to maintain kidney function in the presence of tubular injury detected by NGAL. The equivalent volume of radiocontrast medium indicates a more likely impact of hemodynamic/cardiovascular derangement on the development of AKI, clinical or subclinical.

Elevated NGAL levels were previously shown to be associated with adverse outcomes in STEMI patients [25,26]. The utilization of NGAL in those reports was mainly as a biomarker of an inflammatory response, which may also reflect organ damage or response to coronary artery revascularization. As the current study population did not include patients with cardiogenic shock and chronic kidney disease, the possible implications of elevated NGAL levels in these patients have not been assessed.

We identified a substantial group of STEMI patients who demonstrated subclinical AKI. These patients would have been overlooked using the sCr criteria alone. We further demonstrated that patients with subclinical AKI had a higher risk of adverse outcomes.

Our findings may have important clinical implications. Among patients undergoing PCI, various risk factors were found to be associated with AKI [27]. The detection of this so-called “subclinical AKI” (biomarker elevation in absence of functional AKI) may be identified as a separate clinical entity. It appears that many STEMI patients may experience early episodes of subclinical acute tubular damage. Since NGAL is released from distal tubular segments, including medullary thick ascending limbs, the implication of this study is that there is evidence for medullary injury, in line with the presumed hypoxic nature of tubular injury that specifically involves the outer medulla [28]. CKD may also be present in a patient with normal/increased eGFR values, provided other markers of kidney damage are present for at least three months. It is thus possible that patients with prior myocardial infarction and subsequent renal hypoxia may have chronic kidney disease rather than AKI. Such a perspective modifies other results, because CKD-associated complications include decreased left ventricular function and increased CRP and troponin.

Our study bears notable limitations. This was a single-center study and the number of patients recruited represents a modest sample size. The number of patients included in this work is too small to infer clear associations; however, they suggest the possible role of NGAL in the assessment of mild renal damage.

Our data are based only on measurements of plasma NGAL. The conclusions would have been strengthened if urinary NGAL had been available. NGAL exists in different molecular forms. While monomeric and heterodimeric forms are the predominant forms produced by tubular epithelial cells and represent tubular injury, the dimeric form primarily originates from neutrophils and is elevated in various inflammatory states. As stated above, elevation of NGAL in STEMI may also represent an acute inflammatory response or a response to heart failure.

As plasma NGAL levels are affected by reduced renal clearance, the presence of CKD in some patients may have confounded the results. It appears that further investigation is needed before firm conclusions about the origin of NGAL can be drawn. We have not checked for other biomarkers of proximal tubular injury (such as kidney injury molecule 1), which may aid the differentiation between proximal and distal tubular damage.

We used sCr and eGFR as surrogate markers of kidney function, knowing that these markers have limitations when used in acute hospitalized patients with STEMI.

## 5. Conclusions

Subclinical AKI is common among STEMI patients and is associated with adverse outcomes even in the absence of diagnostic increase in sCr.

## Figures and Tables

**Table 1:** Baseline characteristics of 223 STEMI patients according to AKI state occurrence.

Variable	No AKI	Subclinical AKI	Clinical AKI	*p*-Value
	*n* = 136	*n* = 45	*n* = 42	
Age (years)	61 ± 12	69 ± 13	72 ± 11	<0.001
Men	113 (83%)	35 (78%)	33 (79%)	0.511
Admission systolic blood pressure (mm/Hg)	135 ± 26	130 ± 27	139 ± 33	0.233
Admission diastolic blood pressure (mm/Hg)	83 ± 15	82 ± 16	85 ± 15	0.412
Admission pulse	80 ± 18	78 ± 17	77 ± 15	0.561
Diabetes mellitus	43 (32%)	11 (24%)	15 (37%)	0.467
Dyslipidemia	80 (59%)	26 (57%)	28 (33%)	0.728
Hypertension	62 (46%)	28 (56%)	33 (79 %)	0.004
Chronic kidney disease (any)	5 (4%)	7 (15%)	25 (59%)	<0.001
Chronic kidney disease stage 3b, 4, and 5	1 (1%)	4 (9%)	9 (21%)	<0.001
Smoking history	67 (49%)	20 (44%)	11 (25%)	0.104
Family history of CAD	35 (26%)	5 (10%)	7 (17%)	0.258
Prior myocardial infarction	27 (18%)	13 (22%)	11 (25%)	0.106
No. of narrowed coronary arteries:				0.01
1	54 (40%)	20 (42%)	16 (37%)	
2	46 (34%)	14 (31%)	7 (17%)	
3	36 (27%)	11 (24%)	19 (46%)	
Symptom duration (minutes)	310 ± 285	524 ± 457	653 ± 367	<0.001
Door to balloon time (minutes)	45 ± 25	57 ± 19	59 ± 27	0.318
Baseline C-reactive protein (mg/dL)	12 ± 8	15 ± 10	25 ± 12	<0.001
Baseline eGFR mL/min/1.73 m^2^	91 ± 28	84 ± 25	73 ± 15	<0.001
Baseline serum creatinine, mg/dL	0.84 ± 0.15	0.88 ± 0.19	0.98 ± 0.12	<0.001
Peak serum creatinine, mg/dL	0.88 ± 0.16	1.07 ± 0.32	1.46 ± 0.23	<0.001
Serum creatinine change, mg/dL	0.05 ± 0.04	0.15 ± 0.12	0.47 ± 0.35	<0.001
Contrast volume, mL	147 ± 48	134 ± 47	139 ± 41	0.256

CAD—coronary artery disease; eGFR—estimated glomerular filtration.

**Table 2:** In-hospital outcomes for 223 STEMI patients according to AKI occurrence.

Variable	No AKI	Subclinical AKI	*p*-Value	Clinical AKI	*p*-Value *
	*n* = 136	*n* = 45		*n* = 42	
Length of hospital stay, days	4.1 ± 1.2	5.2 ± 1.1	0.01	4.8 ± 2.9	0.006
Left ventricle EF	47 ± 10	44 ± 10	0.06	41 ± 8	0.007
Left ventricular EF ≤ 45	32 (23%)	15 (33%)	0.01	23 (54%)	0.01
In-hospital adverse outcomes	66 (48%)	33 (73%)	0.005	32 (75%)	0.001
Peak C-reactive protein (mg/dL)	26 ± 28	45 ± 23	0.01	88 ± 75	0.001
Peak Troponin (×10^3^) ng/dL, Median, IQR	20 (50)	39 (71)	0.001	101 (208)	<0.001

* *p* value for patients with clinical AKI vs. no AKI; EF—ejection fraction.

**Table 3:** Multivariate logistic regression model for predictors of in-hospital major adverse outcomes.

	Model 1			Model 2		
	Odds Ratio	95% CI	*p*-Value	Odds Ratio	95% CI	*p*-Value
**Age (years)**	1.02	0.96–1.07	0.605	1.01	0.96–1.07	0.605
**Peak troponin (ng/dL)**	1.000	0.99–1.00	0.489	1.000	0.99–1.00	0.473
**Left ventricle EF**	0.73	0.66–0.82	<0.001	0.74	0.66–0.81	0.001
**Peak CRP (mg/L)**	1.01	1.00–1.03	0.02	1.01	1.00–1.03	0.03
**Any AKI (clinical and subclinical)**	3.83	1.41–11.48	0.01			
**Subclinical AKI**				3.71	1.30–10.62	0.02

CI—Confidence interval; EF—ejection fraction; CRP—C-reactive protein; AKI—acute kidney injury.
